# Inhaled Corticosteroid Exposure and Risk of Cataract in Patients with Asthma and COPD: A Systematic Review and Meta-Analysis

**DOI:** 10.1155/2023/8209978

**Published:** 2023-10-19

**Authors:** Osman Savran, Charlotte Suppli Ulrik

**Affiliations:** ^1^Department of Respiratory Medicine, Copenhagen University Hospital, Hvidovre, Denmark; ^2^Institute of Clinical Medicine, University of Copenhagen, Copenhagen, Denmark

## Abstract

**Purpose:**

Both systemic and inhaled corticosteroids may increase the risk of cataract in patients with both chronic obstructive pulmonary disease (COPD) and asthma. Our aim was to assess the degree of association between cataract and corticosteroid exposure in patients with asthma and COPD.

**Methods:**

A systematic literature review and meta-analysis was performed according to the Preferred Reporting Items for Systematic Reviews and Meta-analyses guidelines. The odds ratio estimates were extracted from each article. A random effects model was applied for estimate pooling in separate meta-analyses according to study design. Meta-regression was performed to assess the dose-response relationship between corticosteroid exposure and the risk of cataract development.

**Results:**

A total of 19 studies met the criteria for inclusion in this review, of which 12 studies provided effect estimates for pooled analyses. All but one of the included observational studies reported a significant association between use of corticosteroids and cataract development in cohorts of asthma and/or COPD patients. Pooled analyses revealed on average a doubled risk of cataract in corticosteroid-exposed asthma and COPD patients. Studies have shown that daily high-dose inhaled corticosteroid (ICS) ≥ 1000 *μ*g is associated with a significant risk of developing cataract and by that predispose to subsequent cataract surgery, although one study showed that systemic corticosteroids increase cataract risk more than ICS.

**Conclusion:**

ICS treatment in asthma and COPD patients is a risk factor for cataract development. Our results emphasize a previously underestimated potential long-term risk of treatment with ICS and underline the importance of targeting ICS treatment, and not least dosing, to improve the risk-benefit ratio of maintenance treatment in both asthma and COPD.

## 1. Introduction

Cataract is a prevalent eye condition characterized by an increasingly opaque lens over time leading to blurred vision [1, 2]. Cataract is the most common treatable cause of acquired blindness worldwide [3]. Eye examination showing the presence of cataract [4] and surgery is the treatment of choice when loss of vision affects daily activities [5, 6].

It has for decades been reported that long-term use of systemic corticosteroids may be associated with the development of cataract. The first study was published almost six decades ago [7]. The type of cataract usually associated with long-term systemic corticosteroid treatment is posterior subcapsular cataract (PSC) [8, 9].

A possible association between cataract development and long-term treatment with inhaled corticosteroids (ICS) for asthma and chronic obstructive pulmonary disease (COPD) has been attracting considerable interest [10–14]. The association has been assessed in a recent review [15]. However, since then, more studies have been published addressing the possible association between cataract development and corticosteroid treatment in asthma and COPD [11, 12, 14, 16–18].

A challenging aspect of both obstructive airways diseases and cataract is that they both cause great morbidity and affect millions of people worldwide [19, 20]. The risk of developing cataract due to ICS treatment for asthma and COPD is most likely lower than cataract risk due to systemic corticosteroid treatment, but this has only been addressed in very few studies [21, 22].

Several studies have proposed a high prevalence of comorbidities among cataract patients, including respiratory diseases such as asthma and COPD [10, 23–26]. Our aim was to explore the possible association of ICS therapy in asthma and COPD and the risk of cataract development. In order to reduce the risk of development of cataract in asthma and COPD patients, it is of great importance for both patients with obstructive airways disease and society to have access to the best-possible knowledge of the risk factors for cataract development.

In this systematic review and meta-analysis, we provide an update on current knowledge regarding the cataract risk in COPD and asthma patients associated with their prescribed systemic and/or inhaled corticosteroids.

## 2. Materials and Methods

### 2.1. Search Strategy

This systematic review was performed and reported in agreement with the Preferred Reporting Items for Systematic Reviews and Meta-Analyses (PRISMA) guidelines [27].

Studies were included if they fulfilled the following inclusion criteria:

- Original research reporting on cataract risk in asthma and/or COPD patients prescribed systemic and/or inhaled corticosteroids
- Studies published within the last three decades

Studies were not included if they fulfilled at least one of the following exclusion criteria:

- Non-English publications
- Systematic reviews

Studies fulfilling the criteria were included in a meta-analysis and meta-regression provided that they reported odds ratio (OR) on the risk of cataract in a sample of patients with asthma and/or COPD treated with inhaled and/or systemic corticosteroid.

A systematic search for recent literature was conducted (last updated March 2023) in the databases of PubMed and EMBASE to identify potentially eligible studies.

The search string on PubMed consisted of the following text words: cataract and corticosteroids.

The EMBASE search was conducted in the Ovid search database using a combination of the following text words “corticosteroid,” “asthma,” and “COPD” and the following subheadings “Diagnosis, Drug Therapy” of the subject heading “cataract.” The final search was conducted by combining all search concepts as the following: “corticosteroids or asthma or COPD and cataract/di, dt (Diagnosis, Drug Therapy).”

### 2.2. Study Selection and Data Extraction

All studies retrieved from the searches in PubMed and EMBASE were screened by both authors (OS and CSU). The final inclusion of a study in the present review was based on consensus between both authors. Studies were screened for eligibility based on the title and abstract.

Data from eligible studies on association between cataract development and corticosteroid treatment for asthma and/or COPD were obtained by the PICO approach for automatic data extraction. Both authors retrieved information from all studies fulfilling the eligibility criteria and performed data extraction. Study details extracted included the following: study title and design, length of study, population, sample size, mean age of the included individuals, definition of the control group, statistical analysis, outcomes reported, and results.

The primary outcome of interest was the assessment of the cataract risk in the included studies. We also screened the studies for the applied definition of asthma and/or COPD and whether included patients had been prescribed inhaled and/or systemic corticosteroid. We retrieved information on daily dose and whether oral corticosteroids were prescribed as a rescue course or as maintenance therapy.

For the development of a pooled effect size estimate in meta-analyses, only odds ratios were used as most of the eligible studies investigating the risk of cataract in asthma and/or COPD patients provided this effect size. Some studies, however, provided risk ratios or hazard ratios. Due to the inclusion of three types of observational studies, that is cohort, cross-sectional, and case-control studies, separate meta-analyses were performed according to the study type.

### 2.3. Quality Assessment

The first author OS performed quality assessment of all included studies. Discrepancies were discussed afterwards between the authors and solved by consensus. All included studies were assessed individually for quality and risk of bias using the standard Newcastle-Ottawa Scale (NOS) for case-control and cohort studies [28] and the adapted NOS for cross-sectional studies [29]. The NOS consists of eight items within three categories. The maximum total is nine, and a study score ≥7 is considered a high-quality study. The adapted version used for the cross-sectional studies consists of seven items within three categories with the maximum total score also being nine.

### 2.4. Data Analysis

Statistical analyses were performed using the Meta-package for RStudio Version 1.2.5001 2009–2019 RStudio, Inc. This package includes the function MetaGen, which provides the generic inverse variance method for meta-analysis [30]. This method is used for pooling of effect sizes such as the risk ratio or odds ratio in precalculated effect sizes based on binary outcome data (i.e., cataract or no cataract development). The output is treatment estimates, standard errors, and confidence intervals as well as an estimate of statistical heterogeneity and tau^2^. The package also includes the function MetaReg, which provides a method for meta-regression. This method incorporates a meta-analysis object and the input of covariate name such as a dichotomous outcome, that is dose-response or no dose-response relationship between corticosteroid exposure and cataract risk in included studies. Sensitivity analysis was performed by excluding each study in a series to establish individual study impact on the pooled odds ratio (OR) estimates. An assessment of funnel plot asymmetry was tested using the Harbord test with a *p* value <0.10 signifying publication bias [31].

## 3. Results

### 3.1. Characteristics of Included Studies

**Figure 1:**
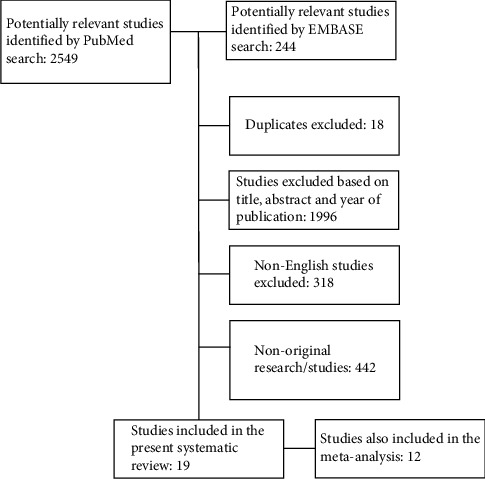
Consort diagram showing the study selection process.

The search algorithm yielded a total of 2,793 hits. The flowchart of the selection process of potentially eligible and included studies addressing a possible association between corticosteroid exposure in patients with asthma and/or COPD and cataract development is given in Figure 1.

A total of 19 studies fulfilled all criteria and were included in the present systematic review, and furthermore, 12 studies (63%) were included in three separate meta-analyses of pooled estimates of odds ratios according to the study type. Studies included in a meta-analysis were either cross-sectional or cohort studies except for two case-control studies. Two observational studies did not comprise effect sizes allowing pooling and were therefore not included in one of the meta-analyses [22, 32]. The most common effect size in the included studies was OR, whereas five studies reported risk ratios (RR) or hazard ratios (HR) and were, therefore, not included in the meta-analysis.

Table 1 summarizes the main characteristics of the studies.

In total, 1,274,878 individuals were included in this systematic review. Of these, 13,343 (1%) and 562,745 (44%), respectively, were included in the meta-analysis of cross-sectional studies and cohort studies, and 25,693 (2%) were included in the meta-analysis of case-control studies. In the systematic review, there were 62,292 (5%) cases of cataract and 996,328 (78%) patients with asthma and/or COPD and prescribed corticosteroids. Of the total number of individuals included in the present systematic review, 642,621 (50%) COPD and/or asthma patients had ever or occasional (at least one 12 months before enrollment [17]) oral corticosteroid (OCS) prescription, 4,054 (0.3%) COPD and/or asthma patients prescribed a combination of both ICS and OCS during the treatment period, 290,891 (23%) COPD and/or asthma patients prescribed or had ever been prescribed ICS.

All but one [36] of the included observational studies reported a statistically significant association between cataract development and corticosteroid exposure in a cohort of asthma and/or COPD patients. The included studies either used clinical examinations such as slit lamp for assessment of lens opacity or diagnostic codes (ICD 374 × [34], ICD-9 code 366 [17, 34], Oxford Medical Information System (OXMIS) [11] or READ codes [11, 16, 18, 36], OSCP4: C71–C75 and ICD 10 code: H25 [14], ICD-10: H25.0–H25.2, H25.8–26.4, H26.8-H26.9, H28.0–H28.2, H59.0, Q12.0 [36]) in medical records to assess the presence of cataract or previous cataract surgery. The included studies all showed that a mean daily dose of ICS exceeding 1,000 *μ*g was associated with a substantial increase in the risk of cataract [25, 26, 33–35].

Four of the included observational studies comprised a cohort of cataract patients (*n* = 123,065) [25, 26, 34, 37] and, similar to the other included studies, assessed the potential association with previous corticosteroid exposure. The remaining studies included asthma or COPD patients prescribed corticosteroid either in a subgroup analysis or in the main population of the study.

Definitions of asthma and COPD were, as expected, different in the included studies. Some studies defined asthma based on questionnaires, including questions on whether the individuals ever had corticosteroid tablets or inhaled corticosteroid, including beclomethasone, budesonide [12, 21, 25, 32], flunisolide, and triamcinolone [35], for asthma. Other studies used a definition based on diagnoses in medical records of COPD or asthma [11, 14, 16–18, 33, 36]. However, one of the included studies did not provide details of the applied definition of asthma and COPD [38]. Two studies reported that the diagnosis of asthma was based on the Global Initiative for Asthma (GINA) recommendations [12, 39]. Studies had obtained details on the use of either oral [11, 12, 16–18, 37–40] or inhaled corticosteroids [14, 25, 26, 32–36] or both [21, 22]. All the included studies focusing on cataract subtypes as the outcome used slit lamp examination for assessment and found that posterior subcapsular cataract (PSC) was the most often found cataract subtype in patients with corticosteroid exposure [21, 22, 25, 32, 37].

### 3.2. Meta-Analysis

**Figure 2:**
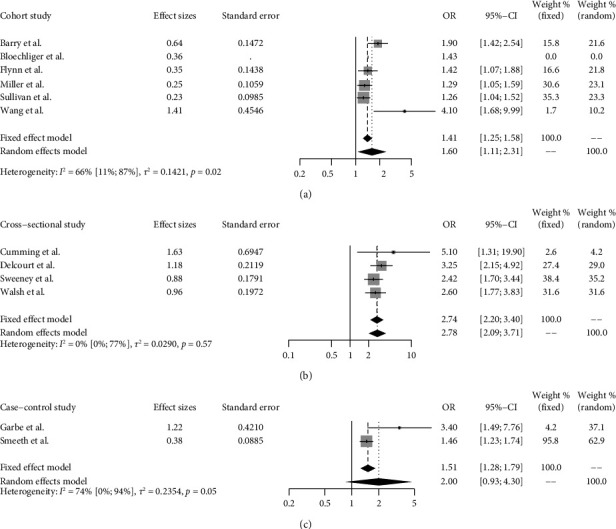
Forest plots depicting the findings from the meta-analysis of (a) cohort, (b) cross-sectional, and (c) case-control studies. Odds ratio (OR) is illustrated as a vertical line. The 95% confidence interval (CI) is illustrated as a line on both sides of the OR. The heterogeneity test was completed to test for any difference between studies. A *p* value <0.05 was defined as statistically significant.

In a pooled effect size estimate analysis of cohort studies, the weighted odds ratio applying a random effects model was 1.60 (95% CI 1.11 to 2.31). Similarly, the weighted odds ratio for cross-sectional and case-control studies were 2.78 (95% CI 2.09–3.71) and 2.0 (95% CI 0.93–4.30), respectively. More details of the findings are given in Figure 2.

### 3.3. Sensitivity Analysis

**Figure 3:**
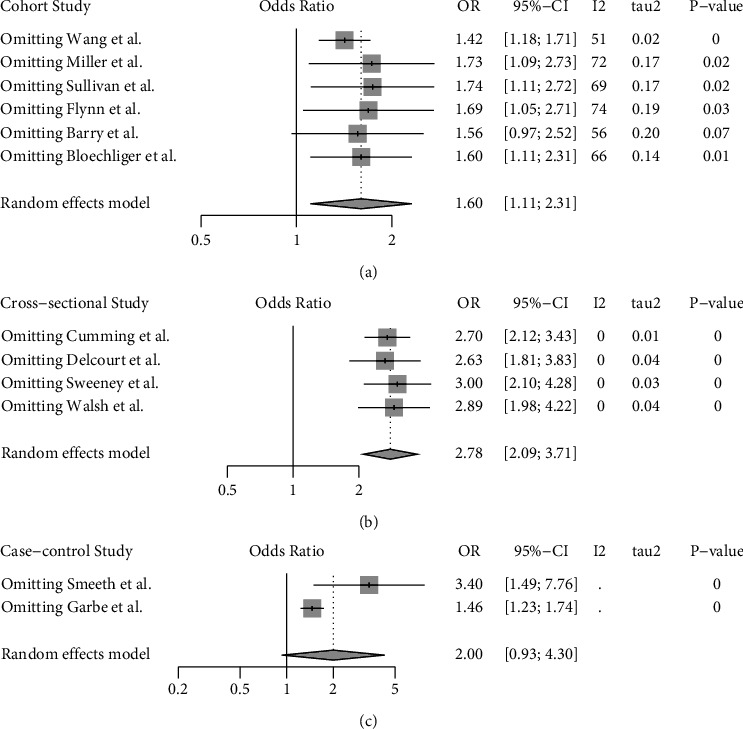
Forest plots depicting the sensitivity analysis in (a) cohort, (b) cross-sectional, and (c) case-control studies. Summary estimates were calculated using a random effects model and by omitting one study at a time. Odds ratio (OR) is illustrated as a vertical line. The 95% confidence interval (CI) is illustrated as a line on both sides of the OR. The heterogeneity test was completed to test for any difference between studies. A *p* value <0.05 was defined as statistically significant.

Pooled effect size estimate analysis according to the study design was repeated in series after stepwise omission of each included study in a sensitivity analysis, which revealed that no individual study had an impact of the estimate of the OR of more than 0.18 points in cohort studies (variation of estimates was 1.42 [95% 1.18–1.71] to 1.74 [95% 1.11–2.72]) and 0.22 points in cross-sectional studies (variation of estimates was 2.63 [95% 1.81–3.83] to 3.00 [95% 2.10–4.28]). Only two case-control studies were included, and individual study's impact on the pooled OR estimates was therefore not assessed for this study design (Figure 3).

### 3.4. Quality Assessment

Quality assessment and risk of bias of the included studies are illustrated in Tables 2 and 3. In the cross-sectional studies, there was a high risk of selection bias in half of the included studies as they provided no information on characteristics of the control group or the comparison group (Table 2). On the contrary, cohort and case-control studies had a low risk of bias for most categories presented in Table 3.

### 3.5. Publication Bias Assessment

**Figure 4:**
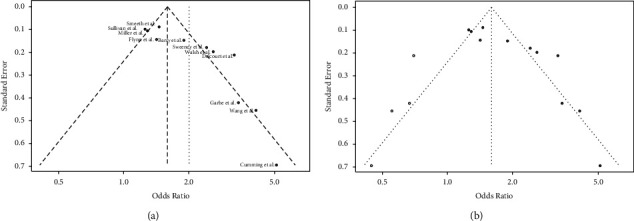
Funnel plot for the assessment of potential publication bias in studies addressing the association between corticosteroid exposure and cataract development. (a) A funnel plot with no additional studies added. Harbod regression test: *t* = 3.55, df = 9, *p* value = 0.006. Including all studies revealed the following effect measurement using a random effects model: OR 2.01, with 95% CI = (1.53–2.63); *p* < 0.001. (b) Represents the trim and fill analysis. Harbord regression test: *t* = 4.33, df = 9, *p* = 0.002. Trim and fill analysis: four studies added on the left side. Harbod regression test: *t* = −0.07, df = 13, *p*-value = 0.948. The following effect measurement using a random effects model was revealed: OR 1.60, with 95% CI = (1.12–2.29); *p* = 0.01. *Note*. Filled circles: observed findings. Open circles: imputed and added studies after trim and fill analysis. The study by Bloechliger et al. was omitted due to missing values.

Included studies demonstrated small study effects as seen by the asymmetric funnel plot, which may be ascribed to publication bias. The Harbord test was statistically significant (*p* < 0.01). The trim and fill analysis found no evidence of publication bias (Figure 4).

### 3.6. Meta-Regression

**Figure 5:**
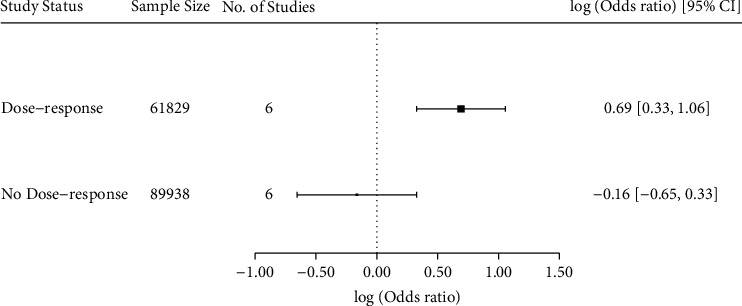
Forest plot illustrating a meta-regression using a random effects model for the assessment of a dose-response association between corticosteroid exposure and cataract development. Included studies were dichotomized on whether they reported a dose-response association between corticosteroid exposure and cataract development. Sigma^2^ = 0.003. Odds ratio (OR) is illustrated as a vertical box. The 95% confidence interval (CI) is illustrated as a line on both sides of the OR.

In a meta-regression of included studies dichotomized according to whether they reported a dose-response association between corticosteroid exposure and cataract development, a dose-response relationship was significantly associated with cataract development (OR 1.99, [95% CI 1.39 to 2.88], *p* < 0.001) (Figure 5).

## 4. Discussion

The findings in this systematic review of available studies suggest a strong association between ICS treatment and the risk of cataract in COPD and asthma patients, with the meta-analysis revealing on average a doubled risk for cataract development in corticosteroid-exposed COPD and asthma patients and the meta-regression revealing a dose-response relationship between corticosteroid exposure and cataract risk. Eight studies reported an increased cataract risk in ICS-treated asthma and COPD patients, and only two of these studies did not adjust for other corticosteroid exposure [21, 22]. The increased cataract risk in COPD and asthma patients is, therefore, not likely influenced by way of corticosteroid administration, and ICS treatment also increases cataract risk.

Almost all included studies using medical records and databases found a significant association between cataract risk and ICS exposure. In contrast, Miller et al. found no significant association between cataract risk and ICS treatment in COPD and were not able to assess the potential impact of corticosteroids administered during hospital admissions [36]. They obtained information from GP's computerized medical records compiled into the General Practice Research Database (GPRD) [36]. Derby et al., Jick et al., and Smeeth et al. on the other hand also used the GPRD and reported a significant association between cataract and individuals with asthma and/or COPD prescribed corticosteroids [26, 33, 38]. This was similar to the study by Derby and Miller who found that there was no increased cataract risk in patients treated with ICS [38]. The study by Jick et al. investigated ICS-treated patients according to daily prescribed doses of less than 500 *μ*g to more than 1500 *μ*g and found an increased cataract risk over a 12-month period [33], while Miller et al. only included patients receiving 10 or more prescriptions for ICS during the study period. Similar to the study by Jick et al., Smeeth et al. also found an increased risk (OR 1.55) of cataract [26]. These results are consistent with our findings, which indicated a dose-response relationship between corticosteroid exposure and cataract risk. In addition, unlike the study by Miller et al. which only included COPD patients, all the other three studies included both asthma and COPD patients. Moreover, all included studies adjusted for age in their analysis and by that reduced the risk of confounding. Some studies also adjusted for treatment with systemic corticosteroid in order to reduce the risk of confounding and further focused on the impact of ICS on cataract development [25, 26, 34, 35].

In the western world, most cases of COPD are caused by tobacco smoking [41]. Besides corticosteroids, tobacco smoke has also been suggested as a potential risk factor for cataract development [42, 43]. Studies included in the present review have also adjusted for smoking history in their analyses to reduce the risk of confounding [25, 33, 36, 38]. In addition, the underlying inflammation in asthma has also been suggested as a risk factor for cataract development [44]. A pathway possibly leading to the development of cataract in patients with allergic asthma has previously been described [44]. However, the potential association between chronic airway inflammation in asthma and the risk of cataract remains to be further investigated.

A systematic review and meta-analysis by Weatherall et al. looking at the association between corticosteroid exposure and cataract was published more than a decade ago [15]. The previous review focused on case-control studies investigating the association between ICS use and the cataract risk and found that use of ICS increased cataract risk by approximately 25% for each 1,000 *μ*g increase in daily dose. The current study also found clear support for an increased cataract risk with daily ICS dose exceeding 1,000 *μ*g. In addition, our meta-regression confirmed a dose-response relationship between corticosteroid exposure and cataract risk. The threshold of a daily ICS dose of at least 1,000 *μ*g suggests a risk of cataract comparable to the risk caused by treatment with systemic corticosteroid [37, 38]. However, the study by Wang et al. showed that the risk of cataract development is almost twice as high in patients treated with systemic corticosteroids compared to patients only treated with ICS [21]. The previous systematic review and meta-analysis was, similar to our study, limited by statistical heterogeneity among the included studies in the analysis [15]. The study by Weatherall et al. did not provide assessment of study quality and bias risk due to the inclusion of only four studies. Given the objective of our study, and, furthermore, the inclusion of a number of study designs, the present study adds important knowledge to the previously published study. In addition, a number of original studies reporting on the association between corticosteroid exposure and cataract development have been published in the last decade, which highlights the need for an update on the current understanding between cataract risk and corticosteroid exposure.

In general, most studies included could be rated as being of high quality (Table 2). However, a few limitations of the included studies are worth mentioning. The study by Toogood et al. and Delcourt et al. did not report how asthma was defined [22, 37]. The study by Derby and Maier, likewise, did not include a definition of COPD [38]. Some of the included studies based on information from medical records relied on a correct diagnosis of COPD and/or asthma and, perhaps, did not confirm diagnosis before enrollment. The study by Cumming et al. and Wang et al. included asthma patients and obtained information from questionnaires regarding corticosteroid treatment for asthma or other chest diseases, which may have led to selection bias [21, 25]. Relatively few asthma subjects were included, which would have made it less likely to detect an association between cataract and corticosteroid treatment [25]. Other included studies used guideline recommendations for both corticosteroid treatment and diagnosis of asthma and COPD. For instance, the study by Ernst et al. used estimates of ICS exposure according to national asthma treatment strategies [34]. Daugherty et al. and Sweeney et al. included patients with severe asthma defined according to the GINA [12, 39]. In addition, it was difficult to assess the overall exposure to both systemic and inhaled corticosteroids as studies only provided relatively few details on the treatment period. Studies either mentioned that patients were prescribed ICS regularly [22], daily [33, 35, 36] or currently [21, 34], or had previously been prescribed OCS [37, 38] or occasionally OCS [17] and did not specify whether it was maintenance treatment and/or rescue courses. The controls in the included studies in most studies had never been prescribed systemic corticosteroid, whereas patients were included provided they had ever had at least one prescription for either ICS or OCS.

### 4.1. Strengths and Limitations

We identified a large number of relevant studies in the past three decades and used, according to guidelines, two reviewers for the selection process, data extraction, and quality assessment of potentially eligible and finally included studies. We provided, unlike the recent review on the association between corticosteroid exposure and cataract development, a meta-regression with a dichotomous outcome, that is dose-response or no dose-response association, and found significant indication of a dose-dependent association between corticosteroid exposure and the cataract risk [15]. The main limitation is that relatively few studies have been published within the last decade, while most of the studies investigating the possible association between corticosteroid exposure and the cataract risk are published some years ago. However, by including a number of study designs, we were able to include more studies and provide independent estimates of the cataract risk based on different populations compared to the previous review [15]. In addition, studies were observational, and assessment of causality is, therefore, difficult, although the inclusion of these studies allowed a more thorough review of the existing literature. The choice of study design could have led to limitations in our systematic review. Though observational studies cover a wide range of studies where the disease of interest occurs spontaneously, they are more prone to bias and confounding. In a systematic review, randomized controlled trials (RCTs) are often preferred instead of observational studies, as they represent the highest level of evidence. They are, however, probably not the best for analyzing long-term side effects of drugs, also due to the majority of studies having relatively short duration. A systematic review and meta-analysis incorporating well-designed observational studies with large populations and sufficient durations could be necessary for a more precise assessment of the relationship between corticosteroid exposure and cataract development. It is also necessary to reduce heterogeneity in methods used for cataract detection and corticosteroid exposure, including daily dose and duration of treatment. Our meta-analysis and meta-regression were limited by heterogeneity of included studies, especially concerning the methods used to detect cataract and the differences in adjustment of variables in the statistical analyses. However, the use of a random effects model allowed for the effect size variation between studies to be taken into account. Furthermore, the asymmetry in study distribution in the funnel plot cannot rule out publication bias due to negative results being difficult to publish. In order to reduce the risk of false-positive results and an appearance of asymmetry in the funnel plot, we used the Harbord test instead of the Egger test as a statistical testing method for funnel plot asymmetry [45]. Moreover, both publication bias assessment and the meta-regression analysis usually demand that the number of included studies exceeds ten. However, the number of included studies was too small for publication bias assessment and meta-regression analysis according to the study design. Both the publication bias assessment and the meta-regression analysis output should therefore be interpreted with caution. We performed both the publication bias assessment and meta-regression analysis by including all studies that reported similar effect measurements on target population and outcome, which could argue for the validity of both analyses. Also, similarly important, the trim and fill method analysis does not include other reasons than publication bias for the asymmetry of the funnel plot, and the method should be interpreted carefully when marked heterogeneity between the included studies exists [46]. Lastly, the different profiles of quality assessment evaluators may have caused difficulties in detecting caveats in study methodology and could be the reason for the high scores on the quality assessment tools. This questions the accuracy of quality assessment and the possibility to clearly identify the risk of bias. However, the tools used for quality assessment and detection of bias risk are validated and provide a comprehensive and systematic assessment of the included studies.

## 5. Conclusion

We have identified several studies revealing an association between corticosteroid exposure and cataract development in asthma and COPD patients. While the included studies mostly agree on the increased risk of cataracts in patients with COPD and asthma, there were biases and limitations associated with the included studies. The most significant findings are that many prescriptions and high daily dose of corticosteroid increase the risk of cataract significantly in both patients with asthma and COPD; that is, a daily dose of 1,000 *μ*g or more of inhaled corticosteroids significantly increases the risk of cataract compared with the risk in patients exposed to a lower daily dose and that patients prescribed oral corticosteroids have a greater risk of cataract development compared to patients prescribed inhaled corticosteroids only. The risk of cataract shown in our meta-analysis should always be weighed against the benefits of ICS in asthma and COPD patients. Cataract screening could, however, be applied for asthma and COPD patients prescribed high-dose ICS treatment in order to reduce the cataract development risk. More research is needed on the best-possible corticosteroid treatment strategies according to treatable traits and other disease characteristics in both asthma and COPD and also to reduce the future burden of cataract.

## Figures and Tables

**Table 1:** Studies examining the association between exposure of (a) inhaled corticosteroids (ICS), (b) oral corticosteroids (OCS), or (c) both ICS and OCS for asthma and/or COPD and the risk of developing cataract.

Author(s)	Objective	Design	Subjects (*n*)	Methods	Results	Adjusted variable	Conclusion
*(a)*							
Cumming et al. [25]	Cataract risk in patients (*n* = 3313) with current and previous ICS use	Cross-sectional study	122 (3.7%) with asthma or other chest illnesses. Mean age 66 years. 1883 females	Slit lamp	PSC OR 5.1 (95% CI 1.3–19.8) with ≥1000 mg overall ICS use (current ICS use OR 3.0 (95% CI 1.7–5.1))	Age, sex, SCS, smoking history, diabetes mellitus, hypertension, sun-related skin damage	Increased ICS dose is associated with cataract development
				Lens			
				Photography	PSC OR 2.7 (95% CI 1.3–5.6) with ≥5 years ICS use		
				Questionnaire on current ICS use, comorbidity, and smoking history			
Jick et al. [33]	Cataract risk in ICS-prescribed COPD or asthma (*n* = 103,289)	Retrospective cohort study	1194 (1.2%) with cataract. Mean age 73 years. 58% females	GPRD	Cataract RR 2.5 (95% CI 1.7–3.6) with ICS prescriptions ≥40	Age, sex, smoking history, diabetes mellitus, hypertension, glaucoma	Higher cataract risk in patients with increasing ICS use
					Cataract RR 2.2 (95% CI 1.3–3.8) with ICS daily dose ≥1000 mg		
Smeeth et al. [26]	ICS associated cataract in patients (*n* = 15,479)	Case-control study	3058 (19.8%) asthma or COPD. Mean age 75 years. 64.6% females	GPRD	Cataract OR 1.32 (95% CI 1.21–1.44) if ever ICS user. OR 1.39 (95% CI 1.26–1.53) for current ICS user. High daily dose ICS (801–1600 *μ*g) OR 1.46 (95% CI 1.23–1.74)	SCS, mean annual consultation rate, ocular or topical corticosteroids, smoking status, BMI, diabetes mellitus, hypertension, glaucoma	If patients had ever used ICS, they had significantly increased odds for cataract development
Ernst et al. [34]	Cataract risk with current ICS and NCS treatment (*n* = 101,805) according to national asthma standards	Prospective cohort study	27,708. Mean age 78 years. Approx. 50% females. ≥4 years follow-up	National health databases	Cataract RR 1.27 (95% CI 1.20–1.40) with daily ICS ≥1000–1500 *μ*g. RR 1.21 (95% CI 1.12–1.31) with NCS >100–200 *μ*g	Topical corticosteroids, allopurinol, major tranquilizers, sex, prior hospitalization, and severity of respiratory disease, OCS, cardiovascular disease, diabetes mellitus, hypertension, rheumatic disease	Significant cataract risk with high-dose ICS
Garbe et al. [35]	Association of average daily ICS dose with cataract extraction in elderly (*n* = 10,214)	Case-control study	3677 with cataract extraction between 1992-1994. 67.4% females. 79.1% ≥ 75 years	Provincial health insurance plan database (RAMQ database) with information on all prescriptions and medical services	Cataract extraction OR 3.06 (95% CI 1.53–6.13) with ICS cumulative use ≥3 years	OCS, age, sex, diabetes mellitus, hypertension, ocular steroids, glaucoma, number of physician claims for service	Significant association between cataract extraction and ICS use
					Cataract extraction OR 3.40 (95% CI 1.49–7.76) with daily dose ICS >1000 *μ*g		
Miller et al. [36]	Daily ICS use and cataract risk in COPD patients (*n* = 53,191)	Prospective cohort study	2941 (5.5%) cataract cases. 51.4% females. Age ≥45 years	GPRD	Cataract OR 1.29 (95% CI 1.05–1.59) with ≥10 ICS prescriptions in the last year	COPD hospitalizations, BMI, smoking status, statin use, depression, diabetes mellitus, rheumatoid arthritis, hypertension, oxygen use, nebulizer use, number of antibiotic prescriptions, concomitant asthma, long-acting beta_2_ agonists, short-acting bronchodilators, inhaled corticosteroid other than fluticasone propionate/salmeterol	ICS exposure not significantly associated with cataract risk
Flynn et al. [14]	Current ICS use and cataract risk in COPD (*n* = 4305)	Cohort study	3243 (75%) ICS exposed. Mean age 65.5 years. 51.9% females	TARDIS database	Cataract OR 1.42 (95% CI 1.07–1.88) with cumulative ICS exposure	Age, sex, social deprivation score, previous ICS, history of OCS, smoking, BMI, primary and secondary cardiovascular disease history, renal failure, COPD severity, history of diabetes mellitus, history of admission with pneumonia	Association between ICS use and increased cataract risk
Nath et al. [32]	Cataract risk and prevalence in COPD (*n* = 405)	Prospective cohort study	COPD *n* = 357. Mean age 64.12 years. 104 (29.13%) females	Screening of ICS treated COPD in attending tertiary care center for tuberculosis and chest diseases	58 (16.24%) cataract cases. Daily ICS dose equivalent to 501–1000 *μ*g fluticasone propionate increased cataract prevalence	Diabetes mellitus, hypertension, other unnamed systemic diseases	Higher cataract prevalence with increasing ICS dose

*(b)*							
Delcourt et al. [37]	Cataract risk factors in patients (*n* = 2468) ever prescribed OCS	Cross-sectional study	153 (6.2%) with asthma. Mean age 70 years. 1386 females	Visual acuity measurement	PSC OR 3.25 (95% 1.39–7.58) with ≥5 years OCS use. OR 2.04 (95% 1.04–3.81) of cataract surgery with ≥5 years OCS use	Age, sex, smoking, diabetes mellitus	Increased cataract risk with increasing OCS prescriptions
				Slit lamp			
Derby et al. [38]	Risk of cataract in patients (*n* = 289,371) with at least one previous OCS prescription	Retrospective cohort study	198,307 (68.5%) with asthma or COPD. Half were ≥50 years. 56% females	GPRD	Cataract RR 4.6 (95% CI 3.7–5.7) with ≥10 OCS prescriptions	Age, sex, general practice, calendar time, prior patient history of hypertension, diabetes mellitus, renal failure, tobacco smoking	Increased cataract risk with increased OCS exposure
				Ophthalmologist referral letters			
Sweeney et al. [12]	Cataract and additional morbidity in patients (*n* = 7,195)	Cross-sectional study	4503 (63%) females. 808 with SA. Mean age 59 years	Database information	Cataract OR 2.42 (95% CI 1.70–3.43) in severe asthma	Hospital admission, age, sex	Significant cataract risk in regularly prescribed OCS in SA
Sullivan et al. [17]	Occasional OCS prescription and association with adverse effects (*n* = 228,436)	Cohort study	72,063 with asthma. Mean age 38 years. 47,293 females	Information from a large insurance claims dataset	Cataract OR 1.26 (95% CI 1.04–1.52) with ≥4 current OCS prescriptions in the past year	Age, sex, geographic region, years since index date, insurance type, use of non-OCS immunosuppressive medication, general comorbidity burden (total number of chronic conditions)	OCS significantly associated with cataract risk
Daugherty et al. [39]	Occasional OCS in severe asthma and cataract risk (*n* = 60,418)	Cohort study	35,424. Mean age 54.8 years. 22,861 (64.5%) females	CPRD	Cataract HR 3.38 (95% CI 2.41–4.73) with a cumulative daily dose OCS ≥7.5 mg	Age, sex, smoking status, diabetes mellitus	Significant cataract risk with high mean cumulative daily dose of OCS
Barry et al. [11]	Regular OCS in severe asthma and cataract risk (*n* = 7195)	Cohort study	808 with SA. Mean age 59 years. 507 (63%) females	OPCRD	Cataract OR 1.9 (95% CI 1.4–2.6) with ≥4 OCS prescriptions in two consecutive years	Age, sex, geographical region	Greater cataract risk with more prescriptions of OCS
Bloechliger et al. [18]	Current, previous, and past OCS use in asthma and cataract risk (*n* = 265,964)	Cohort study	5336. Mean age 72.7 years. 68.9% females	CPRD	Cataract OR 1.43 with ≥4 OCS prescriptions per year and cumulative doses >2000 mg	Alcohol consumption, smoking status, BMI, current or past inhaled bronchodilators use, nonsteroidal anti-inflammatory drugs, platelet aggregation inhibitors, anticoagulants, proton pump inhibitors, vitamin D/calcium bisphosphonates, immunosuppressants, number of ICS prescriptions, Charlson comorbidity index	Increased cataract risk at higher doses and prescriptions of OCS
Price et al. [16]	Adverse events of SCS (98% OCS) treatment in asthma (*n* = 117,409)	Cohort study	Asthma *n* = 48,234 (21%), mean age 49 years. 15,585 (65%) females	OPCRD and CPRD	Significantly increased cataract risk with adjusted HR 1.50 (95% CI 1.31–1.73)	Sex, age, smoking status, BMI, type 2 diabetes mellitus, hypertension, peptic ulcers, depression, cardiovascular disease, antibiotic-treated infections, cumulative inhaled corticosteroid dose, airflow limitation with FEV_1_ < 80%, prior asthma medication use such as short-acting bronchodilators and long-acting beta_2_-agonists	SCS prescribed asthma patients had increased cataract risk
Walsh et al. [40]	Adverse effect of continuous or frequent OCS exposure in asthma and COPD (*n* = 367)	Cross-sectional study	Asthma and/or COPD *n* = 355 (97%), mean age 68 years. 177 (48%) females	Data collection through questionnaires and computerized records from general practices	Significantly increased cataract risk with OR 2.6 (95% CI 1.8–3.9)	Sex, age, BMI, cumulative ICS dose, cigarette consumption, alcohol intake, calcium intake, age at menopause, exercise currently and at age 15–20 years, daily activity	OCS exposure increased cataract risk in asthma and COPD

*(c)*							
Toogood et al. [22]	PSC prevalence in patients (*n* = 400) treated regularly with ICS and OCS	Cross-sectional study	48 (12%) with asthma. Mean age 61. 28 females	Slit lamp	52.1% had lens opacity	Cumulative dose of ICS, age, years of asthma, sex, postmenopausal, arthritis, progesterone-estrogen use	Increased precursor cataract prevalence in ICS and OCS regularly treated patients
Wang et al. [21]	Cataract risk with past or current use of ICS and OCS use in asthma or other diseases (*n* = 3654)	Prospective cohort study	2068. Mean age 63 years. 56% females. Three follow-up examinations	1992–1994, 1997, and 2002:	PSC OR 4.1 (95% CI 1.67-10.08) in current OCS users. OR 2.5 (95% CI 1.33–4.69) in current ICS users	Age, sex, smoking, hypertension, diabetes mellitus, education	High cataract risk with ICS and OCS use
				Lens photography			
				Slit lamp			
				Questionnaires on medical and demographic history. Current and previous medication			

PSC, posterior subcapsular cataract; ICS, inhaled corticosteroid; NCS, nasal corticosteroid; OCS, oral corticosteroid; SCS, systemic corticosteroid; OR, odds ratio; RR, risk ratio; HR, hazard ratio; CI, confidence interval; COPD, chronic obstructive pulmonary disease; GPRD, general practice research database; TARDIS, Tayside allergy and respiratory disease information system; SA, severe asthma; CPRD, clinical practice research datalink; OPCRD, optimum patient care research database.

**Table 2:** Quality and bias risk assessment in included cross-sectional studies.

Cross-sectional studies	Selection				Comparability	Outcome		Score
	Representativeness of the sample	Sample size	Nonrespondents	Ascertainment of exposure	Based on design and analysis	Assessment of outcome	Statistical test	
Toogood et al. [22]	+	−	−	++	+	++	+	7
Mitchell et al. [25]	+	+	−	++	+	+	+	7
Delcourt et al. [37]	+	+	−	++	+	++	+	8
Sweeney et al. [12]	+	+	+	++	+	++	+	9
Walsh et al. [40]	+	+	+	++	+	++	+	9

*Note.* “+” indicates a point. “Ascertainment of exposure,” “comparability,” and “Assessment of outcome” are the only items with a scale of two points. The remaining items are evaluated as zero or one point.

**Table 3:** Quality and bias risk assessment in included cohort and case-control studies.

Cohort studies	Selection				Comparability	Exposure			Score
	Representativeness of the cohort	Nonexposed cohort	Ascertainment of exposure	Outcome of interest is not present at start of study	Based on design and analysis (smoking, sex)	Assessment of outcome	Was follow-up long enough for outcomes to occur?	Adequacy of follow-up of cohorts	
Derby and Maier [38]	+	+	+	+	++	+	+	−	8
Jick et al. [33]	+	+	+	+	++	+	+	+	9
Ernst et al. [34]	+	+	+	+	++	+	+	+	9
Wang et al. [21]	+	+	+	+	+	+	+	−	7
Miller et al. [36]	+	+	+	+	++	+	+	+	9
Sullivan et al. [17]	+	+	+	+	+	+	+	+	8
Daugherty et al. [39]	+	+	+	+	+	+	+	+	8
Flynn et al. [14]	+	+	+	+	++	+	+	+	9
Nath et al. [32]	+	−	−	+	+	+	+	+	6
Barry et al. [11]	+	+	+	−	+	+	+	+	7
Bloechlinger et al. [18]	+	+	+	+	++	+	+	+	9
Price et al. [16]	+	+	+	+	++	+	+	+	9

Case-control studies	Is the case definition adequate?	Representativeness of the cases	Selection of Controls	Definition of Controls	Based on design and analysis (smoking, sex)	Ascertainment of exposure	Same method of ascertainment for cases and controls	Non-Response rate	

Smeeth et al. [26]	+	+	+	+	+	−	+	+	7
Garbe et al. [35]	+	+	+	+	+	+	+	+	8

*Note*. “+” indicates a point. A study can be given a maximum of one point for each item within the “Selection” and “Exposure” categories. A maximum of two points can be given for the “Comparability” category.

## Data Availability

The data used to support the findings of this study are included within the article.
